# Emodin Scavenging of Superoxide Radical Includes π–π Interaction. X-Ray Crystal Structure, Hydrodynamic Voltammetry and Theoretical Studies

**DOI:** 10.3390/antiox9030194

**Published:** 2020-02-25

**Authors:** Miriam Rossi, Kelly Wen, Francesco Caruso, Stuart Belli

**Affiliations:** Vassar College, Department of Chemistry, Poughkeepsie, NY 12604, USA

**Keywords:** superoxide radical, emodin, antioxidant, cyclic voltammetry, ROS

## Abstract

The naturally occurring anthraquinone emodin is found in many plants that have been part of traditional Chinese medicine (TCM) for thousands of years. Recent pharmacological studies suggest that emodin might be a valuable therapeutic option for the treatment of various diseases. We describe the antioxidant effects of emodin on the superoxide radical. Our techniques include X-ray crystallography, density functional theory (DFT), and a recently developed cyclovoltammetry improvement, the rotating ring-disk electrode (RRDE) method. X-ray results show offset π–π stacking of emodin units in the crystal, and this type of interaction is supported by the DFT, which indicates one superoxide interacting via π–π stacking with the quinone moiety, by transferring one electron to the ring, and inducing some quinone aromatization. The second superoxide seems to form a stable complex after interacting with the H(hydroxyl) in position 3 of emodin. We show that one molecule of emodin sequesters two molecules of superoxide: one forming a complex with H(hydroxyl) in position 3, and the other due to π–π oxidation of superoxide and emodin ring reduction. We conclude that emodin is a very strong antioxidant. Color variation in the voltaic cell was observed during the RRDE study. This was analyzed and explained using a mini-grid gold electrode for UV-Vis spectroscopy in the voltaic cell.

## 1. Introduction

Traditional Chinese medicine (TCM) is an ancient set of practices that continues to influence the lives of many health seekers as an alternative to Western medicine. The use of natural, plant-based products in particular, is highly regarded in TCM. One plant that has a long history of usage in TCM is the goji berry (*Lycium barbarum* and *Lycium chinense*). They are widely cultivated in China for their range of health benefits, which include improved liver and kidney tonification and eyesight, elimination of toxins from the body, and an overall improvement in health and longevity [1]. Early goji berry references are from 200 BCE when it premiered as one of the top-grade herbs in Shen Nong Ben Cao Jing, an ancient book detailing Chinese medicinal and agricultural information [1]. Some recent studies show that goji berry has pharmacologic effects, including anti-aging, neuroprotection, immunomodulation, anti-tumor activity, and cytoprotection [2]. Some components of goji berry are E-cinnamic acid, flavonoids such as quercetin, coumarins, lignans, amides, and anthraquinones [3]. Some anthraquinones show activity against constipation, arthritis, multiple sclerosis, and cancer [3]. One of these anthraquinones in goji berry is emodin, also found in rhubarb (*Rheum palmatum*) and other Chinese medicinal herbs. Emodin has shown important biological properties as potent in vitro and in vivo anti-tumor activity, which suggests an important role for treatment of cancer [4]. In addition, emodin sensitizes resistant tumor cells to chemotherapeutic agents such as tumor necrosis factor (TNF)-related apoptosis-inducing ligands, as oxaliplatin, gemcitabine, adriamycin, cisplatin, capecitabine, paclitaxel, abiplastin, doxorubicin, and 5-fluorouracil. [5]. Among other biological effects, emodin prevents calcification stimulated during acute inflammatory response [6]. On the other hand, emodin reverses reactive oxygen species (ROS) in neutrophils derived from severe acute pancreatitis in rats [7]. Moreover, the effect of emodin in fibrotic lung injury is likely related to its favorable properties of anti-inflammation and anti-oxidation. For instance, it stimulates the Nrf2-antioxidant signaling process in damaged lungs [8]. Other biological effects of emodin are attributed to its antioxidant activity including an increase in the hepatic mitochondrial antioxidant condition of CCl_4_-intoxicated mice on emodin pretreatment [9]. In the area of food science, emodin’s antioxidant activity is of interest since anthraquinones, including emodin, were shown to be effective in the inhibition of linoleic acid peroxidation. The results suggest that the emodin antioxidant mechanism possibly involves scavenging of hydroxyl radicals [10].

**Scheme 1:**
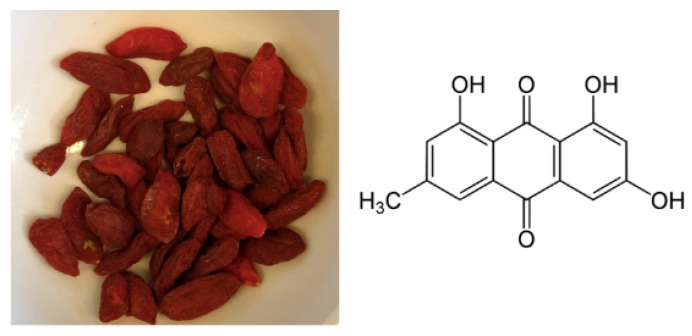
Goji berry and scheme for emodin.

In this study we describe the antioxidant effects of emodin on the superoxide radical, Scheme 1. Our techniques include X-ray crystallography, density functional theory (DFT), and a recently developed cyclovoltammetry improvement, the rotating ring-disk electrode (RRDE) method.

## 2. Materials and Methods

### 2.1. Reagents

DMSO (anhydrous, ≥99.9%), tetrabutyl ammonium bromide (TBAB), and ((2,2-dimethyl-6,6,7,7,8,8,8-heptafluoro-3,5-octanedionato) silver(I)) were purchased from Sigma-Aldrich, St. Louis, MO, USA. DMSO was kept sealed in the original bottle and removed with a syringe in order to protect the solution from atmospheric moisture. Emodin (1,3,8-trihydroxy-6-methyl anthraquinone) was purchased from Sigma-Aldrich and used as received for cyclovoltammetry (CV) study.

### 2.2. Equipment

Hydrodynamic voltammetry at a rotating ring-sisk rlectrode (RRDE) was carried out using the Pine Research WaveDriver 20 bipotentiostat with the Modulated Speed Electrode Rotator. The working electrode is the AFE6R2 gold disk and gold ring rotator tip (Pine Research, Durham, NC, USA) combined with a coiled platinum wire counter electrode and a reference electrode consisting of an AgCl coated silver wire immersed in 0.1 M TBAB in dry DMSO in a fritted glass tube. The electrodes were placed in a 5-neck electrochemical cell together with means for either bubbling or blanketing the solution with gas. Voltammograms were collected using Aftermath software provided by Pine Research. Careful cleaning of the electrodes was performed by polishing with 0.05 µm alumina-particle suspension (Allied High Tech Products, Inc, Rancho Dominguez, CA, USA) on a moistened polishing microcloth to eliminate potential film formation [11].

For spectroelectrochemistry, an HP 8452A Diode Array Spectrophotometer was used to trace the absorbance spectra of emodin when influenced by potential scans in the negative and positive direction. A thin-slit 1-mm quartz cuvette was used as the optically transparent thin layer cell. A gold mini-grid working electrode, Pt counter electrode wire, and Pt reference electrode were used. X-ray diffraction data were collected on a Bruker APEXII diffractometer with MoKα radiation (0.71069 Å) at 125 K.

### 2.3. Superoxide Scavenging by Antioxidants

#### RRDE Study

Stock solutions of emodin 0.1 M in anhydrous DMSO were used in trials. For the experiment, a solution of 0.1 M TBAB in anhydrous DMSO was bubbled for 10 min with a dry O_2_/N_2_ (30%/70%) gas mixture to establish the dissolved oxygen level in the electrochemical cell. The Au/Au electrode was then rotated at 1000 rpm while the disk was swept from 0.1 V to −1.5 V and the ring was held constant at +0.1 V; the disk voltage sweep rate was set to 25 mV/s. The molecular oxygen reduction peak (O_2_ + e^−^→O_2_^−^) was observed at the disk electrode at −0.6 volts; the oxidation current (O_2_^−^→O_2_ + e^−^) was observed at the ring electrode. An initial blank was run on this solution and the ratio of the peak ring current to disk current was calculated as the “collection efficiency” in the absence of an antioxidant. Next, an aliquot of the antioxidant was added, the solution bubbled with the gas mixture for 2 min, and the CV was rerecorded. On again, the reduction and oxidation peaks were measured, and the collection efficiency calculated. Any decrease in the collection efficiency was due to the amount of superoxide removed by the antioxidant. From the known addition of emodin and the amount of superoxide ion removed, the ratio of moles of superoxide scavenged to moles of antioxidant added was calculated.

### 2.4. Computational Study

Calculations were performed using software programs from Biovia (San Diego, CA, USA). Density functional theory code DMol^3^ was applied to calculate energy, geometry, and frequencies implemented in Materials Studio 7.0 (PC platform) [12]. We employed the double numerical polarized (DNP) basis set that included all the occupied atomic orbitals plus a second set of valence atomic orbitals, and polarized d-valence orbitals [13]; the correlation generalized gradient approximation (GGA) was applied including Becke exchange [14] plus Perdew correlation [15] (PBE). All electrons were treated explicitly and the real space cutoff of 5 Å was imposed for numerical integration of the Hamiltonian matrix elements. The self-consistent field convergence criterion was set to the root meanssquare change in the electronic density to be less than 10^−6^ electron/Å^3^. The convergence criteria applied during geometry optimization were 2.72 × 10^−4^ eV for energy and 0.054 eV/Å for force. All calculations included the effect of DMSO as solvent to allow correlation with the experimental features from cyclovoltammetry results.

### 2.5. Diffraction Study

A suitable single crystal of emodin grown in a 1:1 ethanol/water solution was chosen for single crystal X-ray diffraction studies. Data were collected at 125 K; details are given in Table 1. The molecular structure was solved using Bruker-SHELXTL software [16] with refinement of full-matrix least-squares on F^2^; data collection and refinement parameters are summarized in Table 1. Data were deposited at CCDC code 1968867 and available upon request [17]. 

## 3. Results

The X-ray crystal and molecular structure of emodin was solved using data collected at 125 K. Indeed, our structure agrees with the molecular structure of emodin monohydrate reported, (TEVVOG in CCDC) from data at 153 K, whose Rf was 8.04%, [18] compared with ours of 5.91%; this provided standard deviations of 0.003 Å for C–C bonds, more precise than those reported earlier of 0.004 Å [18]. Additionally, our hydrogen atoms were all found experimentally from the Fourier difference electron density. Since hydrogen bonding is of interest in explaining emodin’s mechanism of action, experimentally determined hydrogen atom positions that are freely refined can provide valuable information [19].

**Figure 1:**
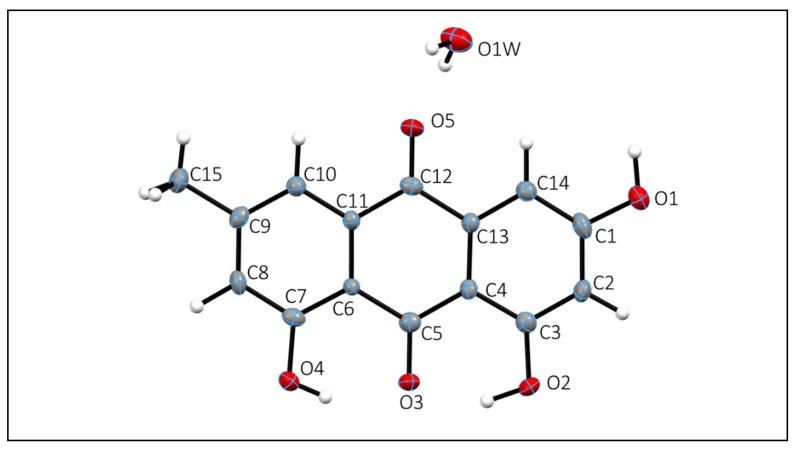
Emodin X-ray structure with atomic labeling. A molecule of water is present.

**Figure 2:**
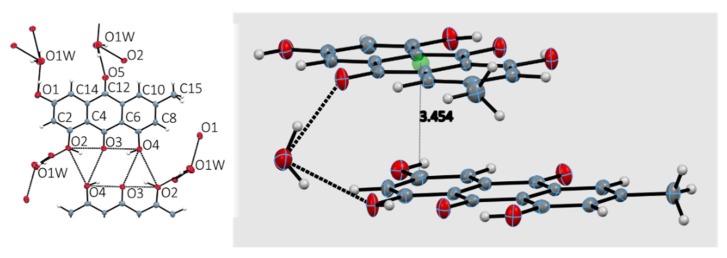
Intermolecular interactions in crystal and H-bonds established between water and aromatic hydroxyls (left). Stacking between emodin rings (right); 3.454 Å is the distance between the quinone centroid (green) and its closest C atom belonging to the lower ring.

Bond distances and angles are consistent with expected values for this type of bonding. The asymmetric unit consists of one emodin molecule and one water molecule (Figure 1). The anthraquinone structure is planar. All oxygen atoms of emodin, including that of the solvent water molecule, are involved in hydrogen bonding either as donors (O–H) or as carbonyl C = O acceptors. The crystal structure also shows π–π stacking (about 3.5 A) between *c*-glide related molecules of the largely planar anthraquinone ring. The packing and intermolecular interactions consist of two emodin molecules that form centrosymmetric hydrogen bond dimers involving the two hydroxyl groups O(2)H and O(4)H and the carbonyl C = O(3) (Figure 2).

In addition to its unique features as a biologically active natural product, emodin also exhibits interesting physical properties relating to its color. The pure compound is a yellow-orange powder and recent studies have shown that emodin exhibits a variety of polymorphic crystal structures that are largely dependent on the solvent used to crystallize the compound. This study in fact shows that different solvate structures exhibit different hydrogen bonding patterns and these patterns can be correlated with the color of the emodin crystals [20].

## 4. Discussion

**Figure 3:**
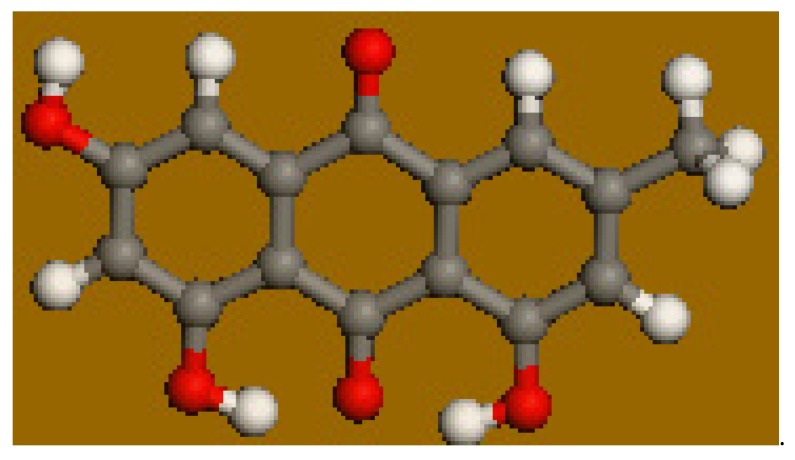
Density functional theory (DFT) of emodin, 9,10-anthracenedione,-1,3,8-trihydroxy-6-methyl, minimum of energy.

**Figure 4:**
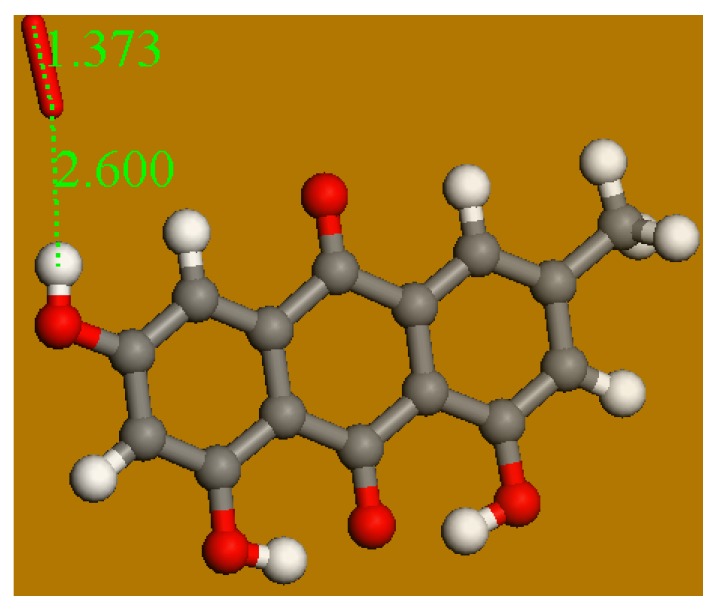
Initial position for superoxide approach to H(hydroxyl) in position 3 of emodin.

Emodin was recrystallized from an ethanol/water mixture and a molecule of water was incorporated in the crystalline cell. However, we did not use these crystals for CV studies, as the water interferes with superoxide. In fact, we used the commercial anhydrous emodin, and high-grade dehydrated DMSO as a solvent in the electrochemical cell. Emodin possesses three hydroxyls and we start the DFT study by eliminating water from the crystal coordinates and proceeding to geometry optimization. The molecule converged showing no imaginary frequencies and so it is at a minimum of energy (Figure 3). Our attempt to capture one of the hydroxyls at positions 1 and 8, located at the bottom of Figure 3, showed no reaction after posing both reagent atoms at van der Waals separation, they did not approach each other. This is due to the strong H-bond between both H(hydroxyl) and the neighbor O(carbonyl), as shown recently by embelin, a related species studied by our group [21]. On the contrary, the hydroxyl in position 3 was susceptible to the superoxide radical attack. Figure 4 shows the initial approach of the superoxide radical (obtained previously with DFT geometry minimization) at van der Waals separation, 2.60 Å, from the H(hydroxyl).

**Figure 5:**
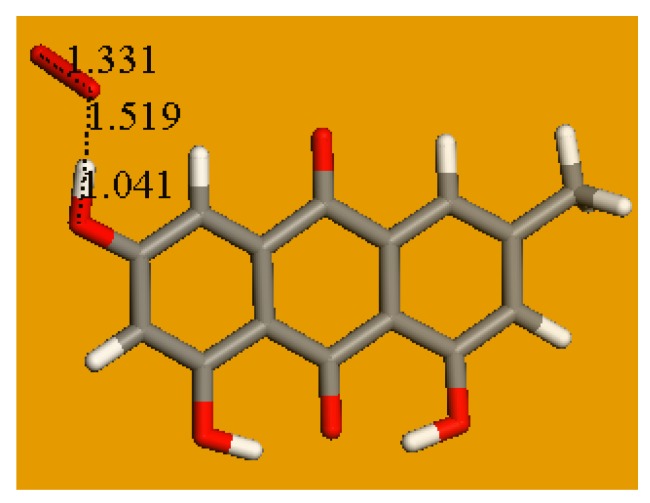
Final position for superoxide approach to H(hydroxyl) in position 3 of emodin.

After geometry minimization, as shown in Figure 5, the potential product for this scavenging was also analyzed with DFT. That is, this arrangement was set for O_2_H**^−^** van der Waals separated from the O(carbonyl) in position 1, and the resulting geometry minimization resulted exactly the same as in Figure 5. Therefore, in this very unusual case the superoxide reached an equilibrium with the antioxidant. It is also feasibly that the three-ring quinone system is not compatible with a radical containing a sort of carbonyl moiety (C8---O), as happens with other scavengers, for instance flavonoids.

**Figure 6:**
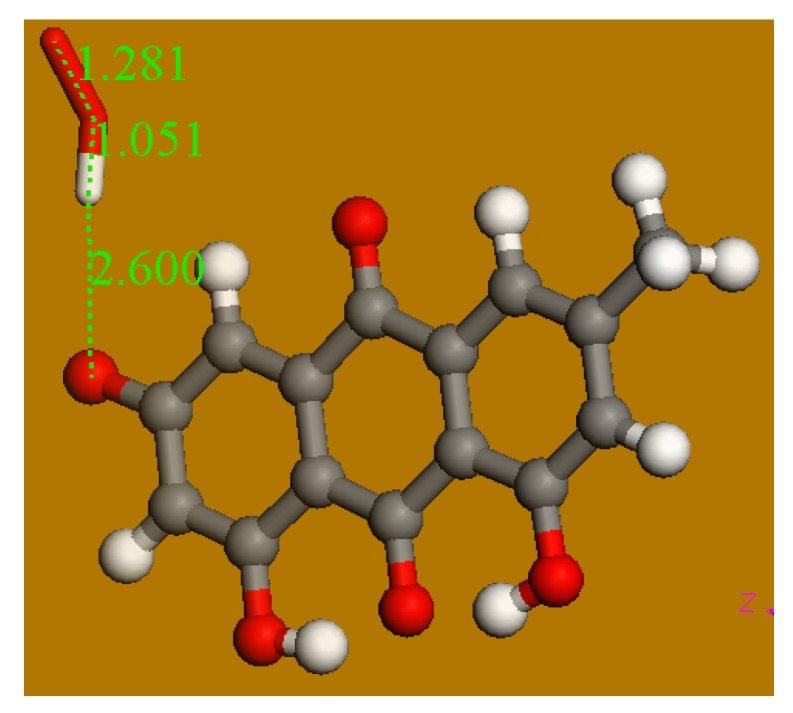
Initial position for O_2_H**^−^** approaching the carbonyl O = C in position 3 of emodin.

**Figure 7:**
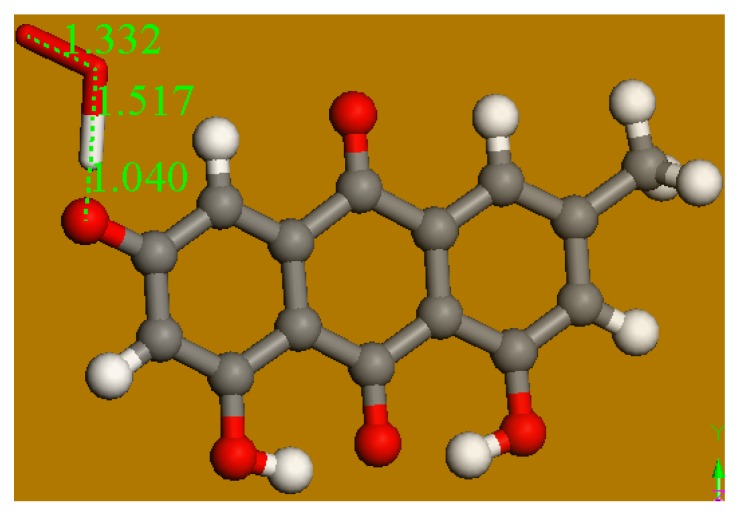
Minimized final position for O_2_H**^−^** approaching the “carbonyl” O = C in position 3 of emodin. H is captured by the macrocycle, demonstrating the impossibility of H abstraction of emodin; we name this sigma scavenging.

An additional analysis was performed for a π–π approaching of superoxide to emodin, as shown in Figure 6 and Figure 7. The product clearly shows a decreased O–O bond distance, 1.259 Å, when compared with superoxide in the initial state, 1.373 Å; meanwhile, the quinone moiety of emodin shows structural changes, for instance, a lengthening of C = C bonds due to the captured electron. The related geometry minimization is characterized by DeltaG = −8.0 Kcal/mol. These results agree with a recent work by our group, where the antioxidant quinone species embelin scavenged superoxide in the same π–π way [21].

**Figure 8:**
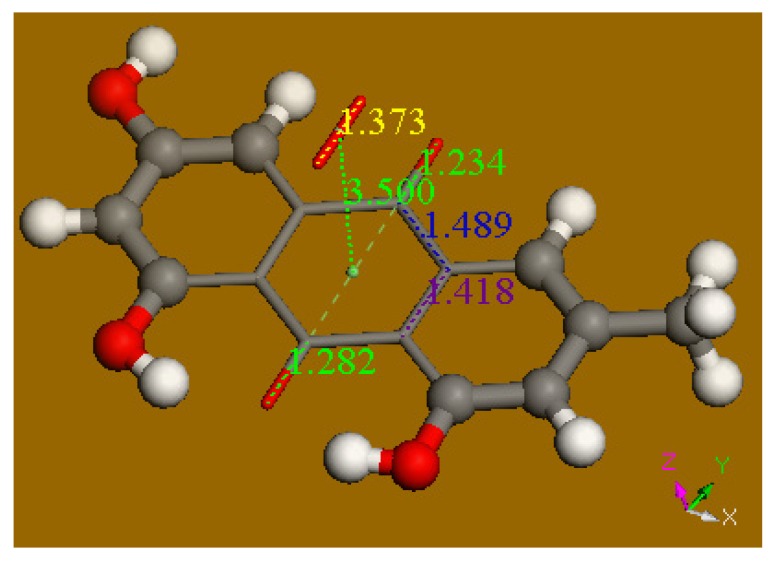
Initial position for superoxide O_2_**-**π–π approaching emodin.

**Figure 9:**
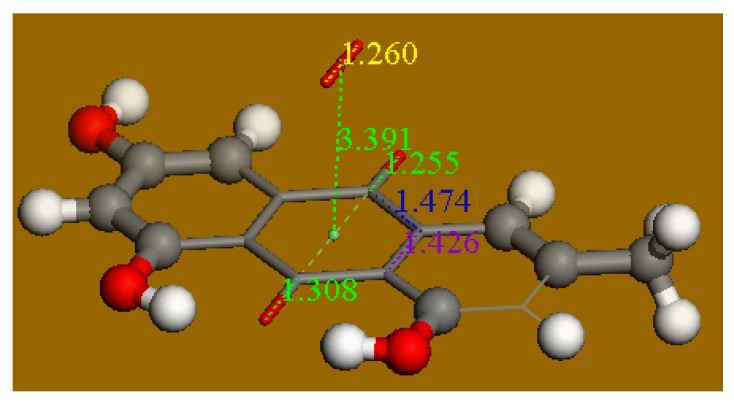
Final position for superoxide O_2_**^.^** π–π approaching to emodin at graphite separation, with associated DeltaG = −8.0 Kcal/mo.l. The d(O–O), 1.260 Å, has decreased from 1.373 Å, getting closer to that in an oxygen molecule (1.227 Å). The separation between the O–O species to the ring, 3.426 Å, is of the same order of graphite stacking distance, 3.35 Å. Marked differences in C–C bond distances in the quinone ring are due to the electron coming from superoxide. This demonstrates that the π–π approach is able to sequester superoxide.

It is therefore interesting to compare precisely the structural parameters at the quinone ring before and after π–π interaction with superoxide (Figure 8 and Figure 9). Carbonyl bond distances d(C = O) of 1.234 Å and 1.282 Å lengthen to 1.255 Å and 1.308 Å, respectively; d(C = C) 1.418 also increases to 1.426, its equivalent specular C = C bond shows the same trend, omitted in Figure 8 and Figure 9 for clarity. In contrast, the single bond distance in the ring decrease, d(C–C) of 1.489 Å becomes shorter, 1.474 Å (the other 3 C–C single bonds, not shown in Figure 8 and Figure 9 for clarity, follow the same trend). Thus, the π–π release of the superoxide electron to the quinone ring induces a sort of aromatization, as initially long (short) bonds become short (long). On the other hand, it is well known that quinones are strong oxidants and this study consistently confirms this property.

Therefore, we can expect two molecules of sequestered superoxide by one molecule of emodin, one forming a complex with H(hydroxyl) in position 3 (Figure 5) and the other due to π–π oxidation of superoxide and emodin aromatic ring reduction (Figure 9). Our study using the RRDE technique is described below to support this theoretical conclusion.

**Figure 10:**
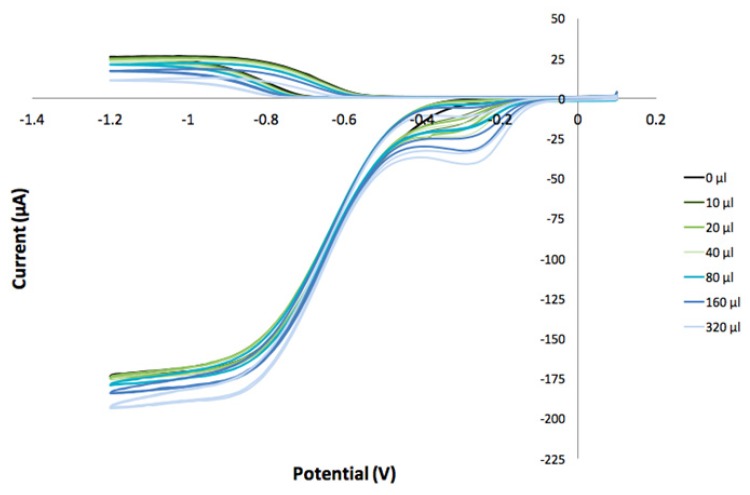
Rotating ring-disk electrode (RRDE) of emodin in DMSO anhydrous solvent.

**Figure 11:**
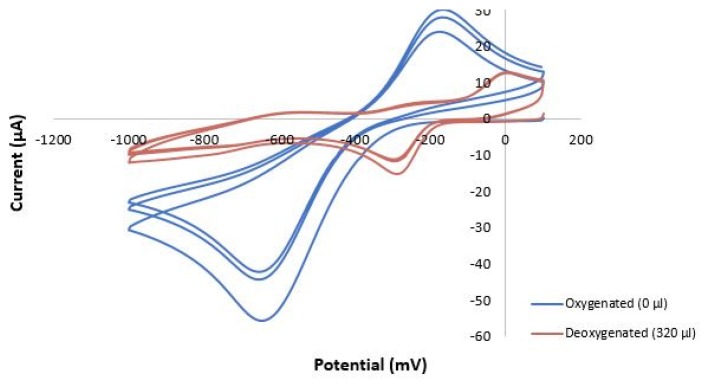
Cyclic voltammograms of oxygenated tetrabutylammonium bromide/DMSO solution (no emodin), showing generation of superoxide, about −0.6 volts, and its reduction, about −0.2 V (blue); and a fixed concentration of emodin, 320 µl, in deoxygenated tetrabutylammonium bromide/DMSO solution (red) showing emodin reduction, about −0.3 V, coincident with the same area of Figure 10.

Figure 10 shows the RRDE voltammograms obtained after addition of aliquots to the voltaic cell containing DMSO bubbled with oxygen. The superoxide is initially generated as reduction of oxygen at the disk electrode, when subjected to sufficient negative potential, lower curve. The opposite reaction, oxidation of superoxide to oxygen, is obtained at the ring electrode, upper curve. Emodin was added in aliquots and the more the antioxidant was added, the less superoxide radical was detected at the ring electrode. In contrast with a previous study [22], there was an increase of current in the area −0.2–0.4 V. Therefore, a specific voltammogram for emodin, without O_2_ in the voltaic cell (e.g., purged with N_2_), was performed (Figure 11) and confirmed emodin reduction in the same area. The redox reaction is not entirely reversible, as indicated on the voltammogram, since the curve generated from the negative scanning is not the same as the curve from the positive scanning (Figure 11). The curves run close but do not overlap entirely. This observation is confirmed by the formation of a “loop” at the end of the disk portion, which is located at the most negative potential.

**Figure 12:**
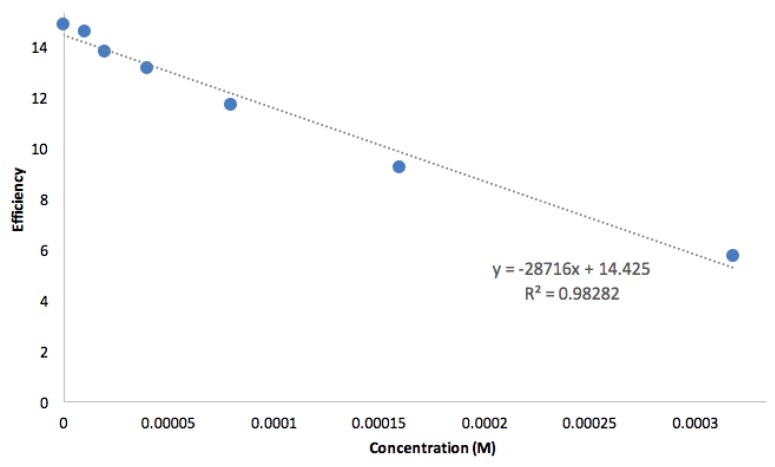
Collection efficiency for superoxide scavenging by emodin through the RRDE technique.

The collection efficiency (Figure 12) is obtained from the voltammogram of Figure 10 by dividing the I value at the ring and that at the disk for each added aliquot, against the concentration of the scavenger emodin in the solution. In a recent work by our group, the slope of the efficiency graph was correlated to the amount of several flavonoid hydroxyls engaged in scavenging the superoxide radical. This resulted in quercetin efficiency slope of −5.30 × 10^4^, steeper than that of chrysin (−1.10 × 10^4^), in correspondence of five hydroxyls of the former and twp of chrysin. According to DFT results, chrysin used only one hydroxyl for scavenging superoxide [22]. The emodin slope (−2.90 × 10^4^) is greater than that of chrysin and smaller than for quercetin. These results agree with the theoretical calculations shown earlier, where two molecules of superoxide were sequestered by emodin; that is, more than chrysin and fewer than quercetin. Indeed, the number and location of hydroxyl groups are believed to affect the scavenging ability of antioxidants [23].

**Figure 13:**
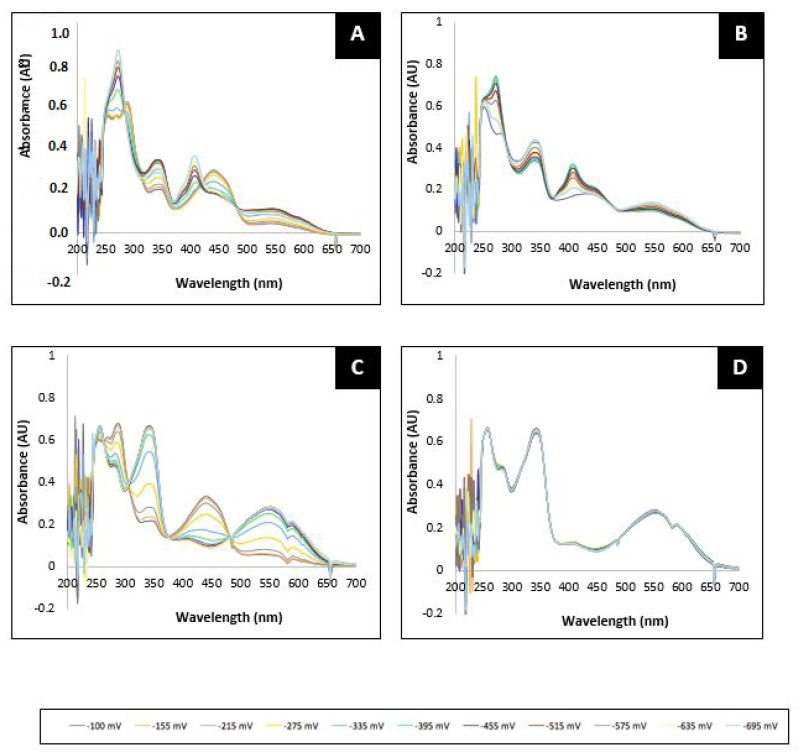
UV-Vis spectra of emodin with and without oxygen. The four experiments consist of bubbling with a gas and a forward conventional CV (−100 mV to −700 mV) or a reverse (−700 mV to −100 mV) scan. (**A**) N_2_ forward scan; (**B**) N_2_ reverse scan; (**C**) O_2_ forward scan; and (**D**) O_2_ reverse scan. It is seen that, for instance, the emodin peak at about 450 nm (A) disappears in (C), suggesting emodin consumed the involving superoxide. Each color represents a specific fixed potential during the UV-Vis experiment. For instance, in (C), containing O_2_, red, the most positive potential, was −0100 V; yellow: −0.215 V; green: −0.335, and so for the visible spectrum increasing negative potential makes peak 450 nm disappear and peak 550 nm increase. On the reversing scan there is no change, and so the “new compound”, associated with peak 450 nm is stable, which may represent the complex of Figure 5.

**Figure 14:**
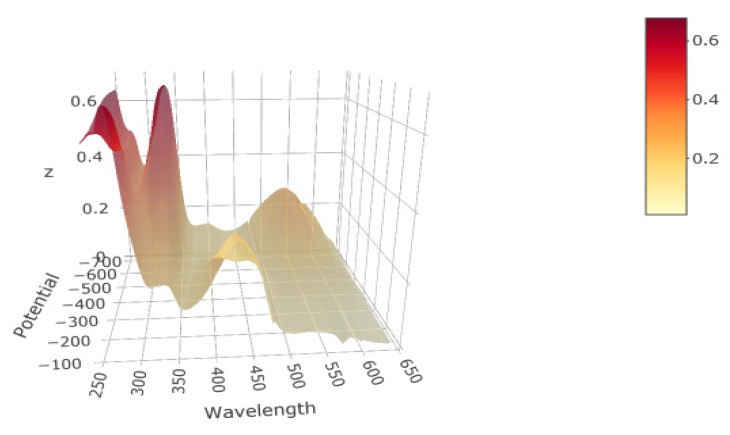
The variation of UV-Vis spectra of Figure 13 is better displayed in this 3D graph. It is shown that peaks 350 nm and 550 nm tend to disappear for more positive potential, meanwhile peak 450 nm increases in intensity. A dynamic 3D graph is deposited [24].

Since change of color was observed during RRDE experiments of emodin, UV-Vis spectra were taken in a voltaic cell using DMSO dry solvent. Figure 13 shows reversible color changes upon the potential changes for the N_2_ bubbled cell, when going to negative potential and vice versa. In contrast, when the O_2_ was bubbled the spectrum became irreversible. There is no straightforward interpretation of these results, but it seems that after O_2_ is trapped sigma style (Figure 5), these molecules follow a different path than what π–π attracted, as the latter should only, at most, regenerate O_2_ and not have important implications. A 3D graph of this experiment is available at the internet [24] and Figure 14 shows a corresponding 2D display.

These results show that emodin is reduced by superoxide in two different ways, sigma and π–π. However, the reduction of emodin by the electrode at negative potential is apparent. Therefore, the slope of emodin indicates not only the electron obtained from superoxide scavenging but also the electron captured from the electrode. The results of all these methods are in agreement and we conclude that emodin is a very good antioxidant.

## 5. Conclusions

This study shows that the rotating ring-disk electrode technique (RRDE) confirms more quantitative results for the determination of antioxidant scavenging when compared with standard CV methods [22]. In addition, emodin is able to scavenge a larger number of superoxide radicals than chrysin and fewer than quercetin, as emodin (slope −2.9 × 10^4^ M^−1^) is weaker than quercetin (−5.3 × 10^4^ M^−1^) and stronger than chrysin (−1.1 × 10^4^ M^−1^) (Figure 12) [22]. In addition to its scavenging of superoxide, emodin is subjected to specific CV reduction, as seen by a bulge around −0.3 V in the RRDE experiment and shows a quasi-reversible cyclovoltammetry. X-ray results show offset π–π stacking of emodin units in the crystal, and this type of interaction is supported by the DFT, which indicates one superoxide interacting via π–π stacking with the quinone moiety, by transferring one electron to the ring, and inducing some quinone aromatization. The second superoxide seems to form a stable complex after interacting with the H(hydroxyl) in position 3 of emodin. Quinones are more active than aromatics at establishing π–π interaction with a radical such as superoxide, and this process is induced by potential electron transfer from the radical species.

## Figures and Tables

**Table 1:** Crystal data for Emodin.

**Chemical formula**	**C_15_H_12_O_6_**	
**Formula weight**	288.25 g/mol	
**Temperature**	125(2) K	
**Wavelength**	0.71073 Å	
**Crystal size**	0.02 x 0.05 x 0.30 mm	
**Crystal habit**	clear orange-yellow needle	
**Crystal system**	monoclinic	
**Space group**	P 2_1_/c	
**Unit cell dimensions**	a = 9.612(2) Å	α = 90°
	b = 15.157(3) Å	β = 113.051(2)°
	c = 9.232(2) Å	γ = 90°
**Volume**	1237.6(5) Å^3^	
**Z**	4	
**Density (calculated)**	1.547 g/cm^3^	
**Absorption coefficient**	0.121 mm^−1^	
**F(000)**	600	
**Theta range for data collection**	2.30 to 28.28°	
**Index ranges**	−12<=h<=12, −20<=k<=20, −12<=l<=12	
**Reflections collected**	26402	
**Independent reflections**	3069 [R(int) = 0.1218]	
**Coverage of independent reflections**	99.9%	
**Absorption correction**	Multi-Scan	
**Max. and min. transmission**	0.9980 and 0.9650	
**Structure solution technique**	direct methods	
**Structure solution program**	SHELXT 2014/5 [16]	
**Refinement method**	Full-matrix least-squares on F^2^	
**Refinement program**	SHELXL-2016/6 [16]	
**Function minimized**	Σ w(F_o_^2^ − F_c_^2^)^2^	
**Data / restraints / parameters**	3069 / 0 / 238	
**Goodness-of-fit on F^2^**	1.709	
**Δ/σ_max_**	0.012	
**Final R indices**	1643 data; I>2σ(I)	R1 = 0.0591, wR2 = 0.0548
	all data	R1 = 0.1390, wR2 = 0.0599
**Weighting scheme**	w=1/[σ^2^(F_o_^2^)] where P=(F_o_^2^+2F_c_^2^)/3	
**Largest diff. peak and hole**	0.404 and -0.327 eÅ^−3^	
**R.M.S. deviation from mean**	0.070 eÅ^−3^	
